# The impact of community- versus clinic-based adherence clubs on loss from care and viral suppression for antiretroviral therapy patients: Findings from a pragmatic randomized controlled trial in South Africa

**DOI:** 10.1371/journal.pmed.1002808

**Published:** 2019-05-21

**Authors:** Colleen F. Hanrahan, Sheree R. Schwartz, Mutsa Mudavanhu, Nora S. West, Lillian Mutunga, Valerie Keyser, Jean Bassett, Annelies Van Rie

**Affiliations:** 1 Department of Epidemiology, Johns Hopkins Bloomberg School of Public Health, Baltimore, Maryland, United States of America; 2 Witkoppen Health and Welfare Centre, Johannesburg, South Africa; 3 Epidemiology for Global Health Institute, University of Antwerp, Antwerp, Belgium; University of Southampton, UNITED KINGDOM

## Abstract

**Background:**

Adherence clubs, where groups of 25–30 patients who are virally suppressed on antiretroviral therapy (ART) meet for counseling and medication pickup, represent an innovative model to retain patients in care and facilitate task-shifting. This intervention replaces traditional clinical care encounters with a 1-hour group session every 2–3 months, and can be organized at a clinic or a community venue. We performed a pragmatic randomized controlled trial to compare loss from club-based care between community- and clinic-based adherence clubs.

**Methods and findings:**

Patients on ART with undetectable viral load at Witkoppen Health and Welfare Centre in Johannesburg, South Africa, were randomized 1:1 to a clinic- or community-based adherence club. Clubs were held every other month. All participants received annual viral load monitoring and medical exam at the clinic. Participants were referred back to clinic-based standard care if they missed a club visit and did not pick up ART medications within 5 days, had 2 consecutive late ART medication pickups, developed a disqualifying (excluding) comorbidity, or had viral rebound. From February 12, 2014, to May 31, 2015, we randomized 775 eligible adults into 12 pairs of clubs—376 (49%) into clinic-based clubs and 399 (51%) into community-based clubs. Characteristics were similar by arm: 65% female, median age 38 years, and median CD4 count 506 cells/mm^3^. Overall, 47% (95% CI 44%–51%) experienced the primary outcome of loss from club-based care. Among community-based club participants, the cumulative proportion lost from club-based care was 52% (95% CI 47%–57%), compared to 43% (95% CI 38%–48%, *p =* 0.002) among clinic-based club participants. The risk of loss to club-based care was higher among participants assigned to community-based clubs than among those assigned to clinic-based clubs (adjusted hazard ratio 1.38, 95% CI 1.02–1.87, *p =* 0.032), after accounting for sex, age, nationality, time on ART, baseline CD4 count, and employment status. Among those who were lost from club-based care (*n =* 367), the most common reason was missing a club visit and the associated ART medication pickup entirely (54%, 95% CI 49%–59%), and was similar by arm (*p =* 0.086). Development of an excluding comorbidity occurred in 3% overall of those lost from club-based care, and was not different by arm (*p =* 0.816); no deaths occurred in either arm during club-based care. Viral rebound occurred in 13% of those lost from community club-based care and 21% of those lost from clinic-based care (*p =* 0.051). In post hoc secondary analysis, among those referred to standard care, 72% (95% CI 68%–77%) reengaged in clinic-based care within 90 days of their club-based care discontinuation date. The main limitations of the trial are the lack of a comparison group receiving routine clinic-based standard care and the potential limited generalizability due to the single-clinic setting.

**Conclusions:**

These findings demonstrate that overall loss from an adherence club intervention was high in this setting and that, importantly, it was worse in community-based adherence clubs compared to those based at the clinic. We urge caution in assuming that the effectiveness of clinic-based interventions will carry over to community settings, without a better understanding of patient-level factors associated with successful retention in care.

**Trial registration:**

Pan African Clinical Trials Registry (PACTR201602001460157).

## Abstract 2

In a randomized trial done in Johannesburg, South Africa, Colleen Hanrahan and colleagues study retention in HIV care at clinic- and community-based adherence clubs.

## Author summary

**Why was the study done?:**

- Adherence clubs are an intervention to retain people living with HIV on antiretroviral therapy (ART) and decongest busy clinics in high-burden settings.
- In adherence clubs, groups of 20–30 patients meet every other month for a 1-hour counseling and medication pickup session in a clinic or community setting.
- There is currently no evidence base to support whether clinic- or community-based adherence clubs are a preferable implementation strategy—community-based clubs may offer closer proximity to patients’ homes, while clinic-based clubs may offer easy access to additional healthcare services, and either model may impact HIV-related stigma perceived or experienced by patients.

**What did the researchers do and find?:**

- We randomized stable ART patients at a clinic in Johannesburg, South Africa, to receive either a clinic- or community-based adherence club intervention, and followed participants for 24 months to determine which type of adherence club led to fewer individuals being lost from care in the intervention.
- Participants in clinic-based clubs had lower levels of loss from the club intervention than those in community-based clubs.
- Overall loss from either type of adherence club-based care was high, with approximately half of individuals dropping out.

**What do these findings mean?:**

- In this study, we found that community-based adherence clubs did not work as well as clinic-based clubs at retaining patients within the club intervention; however, with half of the individuals dropping out of the clubs overall, neither intervention may be a good choice for implementation.
- It is important to understand the mechanism by which individuals are lost from differentiated care interventions such as adherence clubs so that their implementation can be optimized.

## Introduction

World Health Organization (WHO) recommendations for universal antiretroviral therapy (ART) for all people living with HIV regardless of level of immunosuppression have been recently implemented across many high-HIV-burden countries [1,2]. Although intended to provide health benefits at the individual level as well as reduce population-level HIV transmission, this policy may also have the unintended consequence of overburdening already taxed or weak health systems, particularly in low-resourced, high-burden settings. Adherence clubs for clinically stable ART patients have been implemented in some settings to promote task-shifting to lower skilled healthcare workers in order to allow clinicians to handle more complex patients such as those newly initiating ART and those with more complex needs (e.g., comorbidities) [3]. Adherence clubs are groups of 20–30 patients who meet for counseling and ART medication pickup; club visits last approximately 1 hour and occur every 2 to 3 months, with patients also annually having an individual clinician consultation. In addition to decongesting busy clinics, adherence clubs represent a streamlined care experience for people living with HIV that reduces the time spent accessing care [4–6]. Three large observational cohort studies have demonstrated that adherence clubs promote retention in care and viral suppression compared to the clinic-based standard of care [7–9].

Adherence clubs have been implemented within the community setting, where clubs are held at venues such as churches, libraries, or community centers [6–8], as well as within the primary health clinic setting (as in South Africa) [1]. Community-based models of HIV care are recommended by WHO [2], and have been demonstrated to deliver at least comparable outcomes to clinic-based standard of care for clinically stable ART patients in low- and middle-income countries [10]. However, there is currently no published evidence base as to whether community- and clinic-based adherence clubs offer comparable effectiveness in terms of retention in care and viral suppression, nor whether one is more acceptable to patients. Factors such as stigma, convenience, cost, and access to other healthcare services could all be at play. The goal of this study was to compare the effectiveness of community- versus clinic-based adherence clubs with respect to loss from club-based care and viral suppression. We hypothesized that participants in community-based adherence clubs would have lower levels of loss from club-based care compared to those in clinic-based clubs.

## Methods

### Trial design

We conducted a pragmatic, open-label, parallel randomized controlled trial comparing community- versus clinic-based adherence clubs for stable ART patients in Johannesburg, South Africa. Eligible patients were randomized 1:1 to receive assignment to either a community- or clinic-based adherence club, stratified by area of residence.

### Study setting and participants

**Fig 1:**
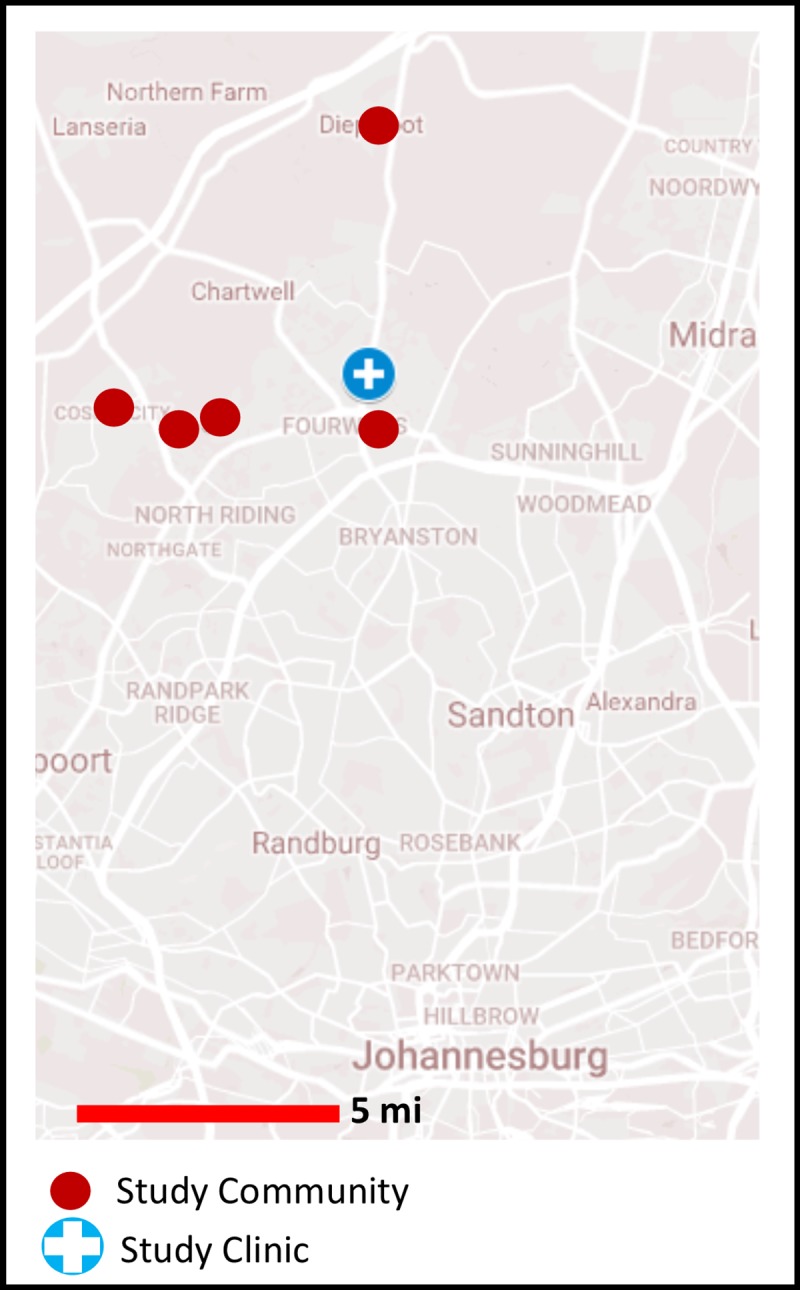
Map of study communities and clinic. Map showing location of study communities (red circles) as well as the study clinic, Witkoppen Health and Welfare Centre (blue circle with cross).

This trial was set at Witkoppen Health and Welfare Centre, a primary care clinic that provides services to approximately 2,000 patients on ART per month, in northern Johannesburg, South Africa. Participants were recruited from patients receiving ART at the clinic. The medical files of those on ART were prescreened daily by a lay HIV counselor in order to identify potentially eligible participants residing in the clinic catchment areas of Diepsloot, Fourways, Kya Sands, Cosmo City, and Msawawa (Fig 1). These individuals were further screened for eligibility by clinicians.

Inclusion criteria were the following: (a) age ≥ 18 years, (b) on the same ART regimen for ≥12 months (or with a regimen change ≥6 months prior to screening where the regimen was changed from zidovudine to tenofovir, from nevirapine to efavirenz, and/or from stavudine to zidovudine or tenofovir, and whose most recent creatinine level was normal), and (c) virally suppressed for at least 12 months (2 most recent viral load results were ≤400 copies/ml). Exclusion criteria were the following: (a) currently on a stavudine-containing regimen, (b) currently pregnant or intending to become pregnant within 6 months of club start, (c) current comorbidity or chronic illness that requires routine, frequent, or complex clinical management (e.g., diabetes, epilepsy, active tuberculosis [TB], cancer, or mental illness), (d) hypertensive and requiring treatment with more than 1 antihypertensive agent, and (e) attending ART care with an HIV-positive child.

Study recruitment was conducted on a monthly rolling basis, and 1 pair of clubs (1 clinic-based and 1 community-based) was launched with newly recruited participants per month. For each month of recruitment, a geographic target area of residence was predetermined. Eligible patients residing within the target area were approached for study enrollment. Those living outside the monthly target area were entered into a database and contacted for recruitment in an upcoming month in which their area of residence was scheduled to be targeted. Patients who agreed to participate and provided informed consent had a baseline blood draw to confirm viral load suppression prior to participation in the first adherence club visit. Individuals who had a baseline viral load of >200 copies/ml were excluded from the study as screening failures.

### Intervention

The adherence club intervention followed the model proposed by Luque-Fernandez et al. [9]. Each club had a minimum of 25 patients and a maximum of 30 patients, was run by a lay HIV counselor, and met every other month (the same interval between appointments in the clinic standard of care). Once annually, the club visit was replaced by a visit to a medical doctor at the clinic. Participant attendance was recorded in a register. Each participant attending a club was weighed and screened for current TB symptoms (current cough of any length, current fever, unintentional weight loss, and/or night sweats), and those experiencing any TB symptom were referred to the clinic for further evaluation. Hypertensive participants had their blood pressure measured at the start of each club visit, and any participant with an abnormal reading (systolic pressure > 140 mm Hg or diastolic pressure > 100 mm Hg) had the measure repeated at the end of the club visit. Participants with 2 abnormal blood pressure readings at the same club visit were referred for evaluation at the clinic. Participants met briefly as a group to discuss an adherence- or ART-related topic, led by the counselor. Each club visit lasted approximately 1 hour. At the end of the club visit, each participant received their 2-month prepacked supply of ART medication (and hypertension medication if applicable). Blood draws for viral load and medication rescripting were conducted every 6 months during the club visit by a nurse. A repeat blood draw was taken if viral load was 50 to 400 copies/ml. Table 1 depicts the schedule of club activities by visit.

**Table 1:** Adherence club visit and activity schedule.

Year	Visit	Month	Visit type	Activities
**Year 1**	0	−1	Recruitment & screening	Consent, baseline blood draw
	1	0	Club visit at clinic	Routine
	2	2	Club visit	Repeat blood draw if needed
	3	4	Club visit	Routine
	4	6	Club visit	Re-scripting
	5	8	Club visit	Routine
	6	10	Club visit	Blood draw
	7	12	Medical visit at clinic	Medical review & re-scripting
**Year 2**	8	14	Club visit	Repeat blood draw if needed
	9	16	Club visit	Routine
	10	18	Club visit	Re-scripting
	11	20	Club visit	Routine
	12	22	Club visit	Blood draw
	13	24	Medical visit at clinic	Medical review & re-scripting

Participants could have a “buddy” pick up their medication at a club visit. The buddy was preselected by the participant and documented upon enrollment. The counselor in charge of the club verified the buddy identification. Participants who were unable to attend a club visit were allowed to pick up their medication at the clinic within 5 days of the club visit on a limited basis (see below).

Clinic-based clubs were held at an onsite meeting space separate from where clinical exam rooms were located. Community-based clubs were held at community venues within the preselected area of residence, including community-based nongovernmental organization facilities, churches, and community centers. These venues were typically 3–5 kilometers closer to the community than the Witkoppen clinic, with the exception of the venue in the Fourways community, which was located a similar distance from the community as the clinic (see Fig 1). The first club visit for community-based clubs was held at the clinic, and all subsequent visits (except for the annual medical visits) were held at the community-based venue.

Reasons for referral from club-based care to standard care (at the clinic) included the following: voluntary return, 2 consecutive buddy pickups in a row, 2 consecutive late pickups (at the clinic instead of at the scheduled club visit), 3 late pickups in 12 months, missing a medication pickup entirely (not presenting at the club visit, sending a buddy, or coming to clinic within 5 days), becoming pregnant, TB diagnosis, requiring treatment with more than 1 antihypertensive agent, identification of an excluding comorbid or chronic condition (at the annual medical visit or any other clinical encounter), ART regimen change for any reason, and viral rebound (defined as 1 viral load measurement of >400 copies/ml or 2 measurements of 50–400 copies/ml). Participants who were referred back to standard care were contacted in order to book a clinic appointment. Those who could not be contacted were referred for defaulter tracing according to the clinic standard of care (3 attempts to contact during different days/times of day).

Participants requiring clinical follow-up (e.g., abnormal blood pressure) were referred to a dedicated nurse at the clinic, who also managed club-based blood draws, ART re-scripting, and annual medical visits. The annual medical visit included review of blood results (viral load, creatinine, alanine transaminase, aspartate transaminase, and CD4 count) and measurement of weight, blood pressure, hemoglobin, and urine glucose, as well as a routine clinical exam and Pap smear for female participants.

### Outcomes

The primary outcome was loss from club-based care, defined as referral to clinic-based standard care for any of the above specified reasons. Participants were assessed for the outcome at each club visit, each annual medical visit and any interim clinical visit made between medical visits. The primary outcome was assessed through review of the club register and review of the participants’ clinical files and electronic medical records.

Key prespecified secondary outcomes were the proportion of patients who voluntarily chose to return to clinic-based standard care, the proportion of patients with medical contraindication for continuation of club-based care (those referred back to clinic-based care because of pregnancy, TB diagnosis, hypertension, identification of an excluding comorbid or chronic condition, or ART regimen change), and all-cause mortality. Participants were followed for outcomes for 24 months following the initial treatment assignment, and outcomes were compared by arm.

A post hoc secondary outcome was the proportion of participants who were referred to clinic-based standard care who reengaged in care by 90 days following referral. We also explored the proportion lost to any ART (inclusive of both club-based care and clinic-based standard care). Engagement in care was assessed using review of routine clinical files and electronic medical records.

### Sample size

We assumed 90% power and a 2-sided α of 0.05. With equal sample size in the 2 arms, using a difference of proportions, we calculated that we would be able to observe a difference in the primary outcome of at least 10% with a sample size of at least 600 individuals (300 per arm), which corresponded to enrolling 12 clubs, each with a minimum of 25 participants per club (see S1 Text for full description of power calculations).

### Randomization

Eligible participants were consented for study participation by a lay HIV counselor. Randomization was performed in a separate room by a research assistant. Prior to the study start, an equal number of assignment sheets were printed (400 per arm). Each sheet was sealed in an opaque envelope, and the envelopes were thoroughly mixed and stored in a locked cabinet in a locked room to which only study staff had access. After consenting, in the presence of a research assistant, each participant chose an envelope and was assigned to the arm indicated inside. The research assistant documented the assignment.

### Statistical methods

We used standard descriptive statistics to characterize the cohort. We used a multivariable Cox proportional hazards model clustered by club to compare participants by arm on our primary outcome of loss from club-based care, adjusting for differences in baseline covariates as well as the prespecified variables age, sex, nationality, and time on ART. We constructed Kaplan–Meier curves of the cumulative proportion retained in club-based care by arm and compared them using the log-rank test. We used differences in proportions to compare reasons for return to clinic-based standard care by arm. We used multivariable linear regression clustered by club to estimate a post hoc secondary outcome of adjusted risk difference. We used a univariate and multivariable Cox proportional hazards model clustered by club, and as well as Kaplan–Meier curves, to compare participants by arm on our post hoc secondary outcome of loss from any ART care. All statistical analyses were conducted using Stata 13. The significance level was set at 0.05.

### Ethics and registration

This trial was approved by the Human Research Ethics Committee at the University of the Witwatersrand in South Africa (Clearance number M131121) and the Institutional Review Board at the University of North Carolina (Approval number 13–3900). The Institutional Review Board at the Johns Hopkins Bloomberg School of Public Health provided authorization to rely on the Human Research Ethics Committee at the University of the Witwatersrand for review and continuing oversight of this trial. All participants provided informed written consent. The trial was registered with the Pan African Clinical Trials Registry (PACTR201602001460157) after the end of participant enrollment (on February 6, 2016) as the authors were not aware of the requirements for registration of behavioral intervention trials. The full protocol for this trial can be accessed in S1 Text.

## Results

### Screening and enrollment

**Fig 2:**
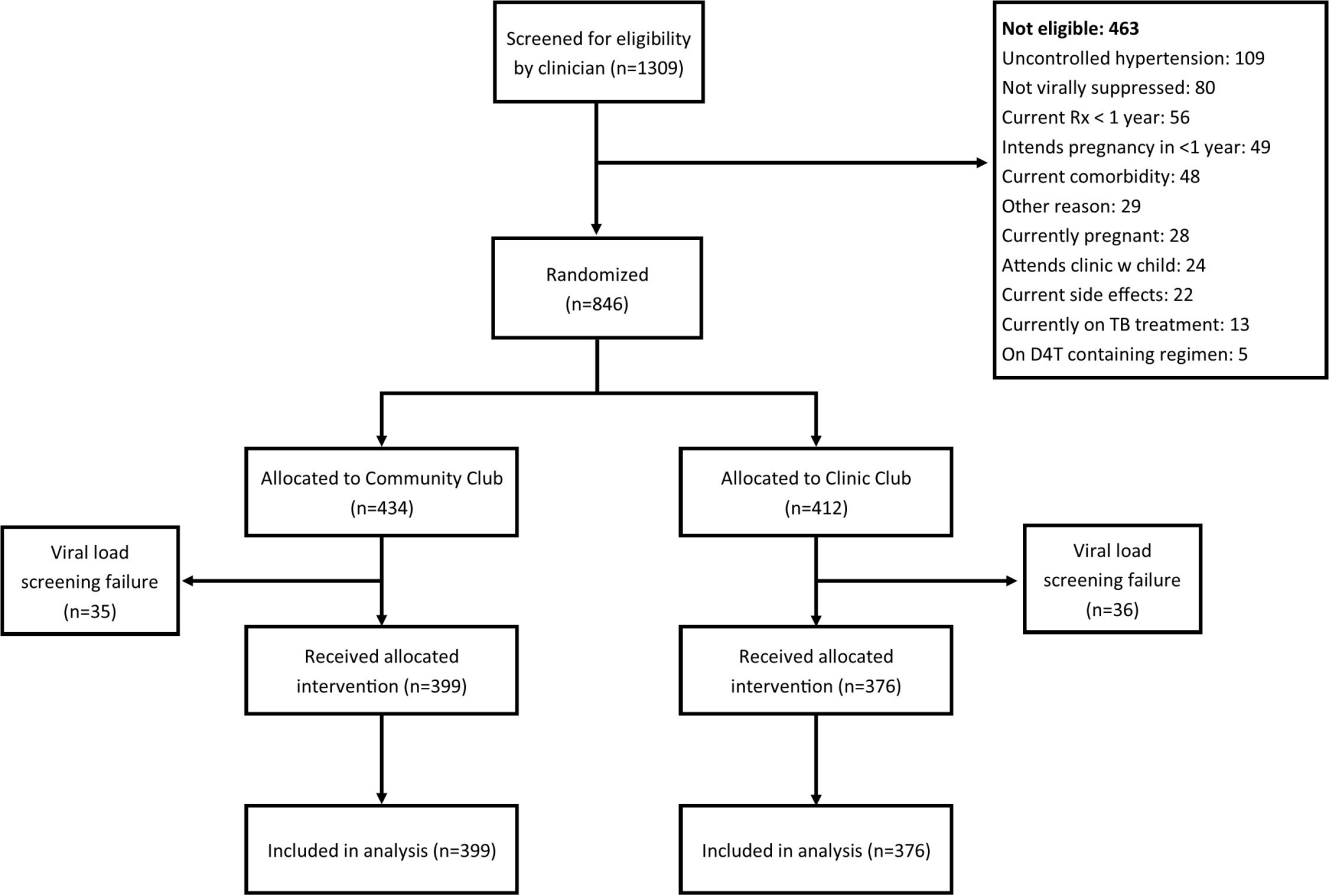
CONSORT diagram of study eligibility screening, randomization, and analysis. D4T, stavudine; Rx, prescription; TB, tuberculosis.

Between February 12, 2014, and May 31, 2015, clinicians screened 1,309 ART patients for study eligibility (see Fig 2). Among those screened, 463 (35%) were ineligible. The main reasons for ineligibility were uncontrolled hypertension (109/1,309, 8%), not being virally suppressed (80/1,309, 6%), and being on current ART regimen for less than 1 year (56/1,309, 4%). A total of 846 individuals were randomized to a community-based adherence club (*n =* 434/846, 51%) or a clinic-based club (*n =* 412/846, 49%). After baseline HIV viral load testing, an additional 71 individuals were identified as ineligible because they were not virally suppressed, representing 8% of those randomized to a community-based club (35/434) and 9% of those randomized to a clinic-based club (36/412). After exclusion of these individuals, there were a total of 775 participants in the study, 399 (51%) in community-based clubs and 376 (49%) in clinic-based clubs, all of whom are included in this analysis.

### Participant and club characteristics

Twelve pairs of clubs (1 community-based and 1 clinic-based club in each pair) were started based on residential area, with more clubs started in areas with a greater density of potential participants. Seven pairs were started for Diepsloot, 3 for Cosmo City, 1 for Kya Sands/Msawawa, and 1 for Fourways. The majority (65%) of club participants were female (Table 2). At baseline, the median age was 38 years (IQR 33–43). More participants assigned to community-based clubs were unemployed (24%, 95% CI 20%–29%) compared to those assigned to clinic-based clubs (17%, 95% CI 13%–22%). The majority of club participants (89%) were currently on a fixed-dose combination of efavirenz, tenofovir, and lamivudine. Individuals with hypertension controlled by 1 antihypertensive agent made up 6% of the participants. The median CD4 count was 472 cells/mm^3^ (IQR 342–665) among those assigned to community-based clubs and 527 cells/mm^3^ (IQR 377–690) among those assigned to clinic-based clubs.

**Table 2:** Baseline participant sociodemographic and clinical characteristics by treatment arm (*n =* 775).

Characteristic	Community-based clubs (*n =* 399)	Clinic-based clubs (*n =* 376)
Female sex	267 (67%)	239 (64%)
Age, median (IQR)	38 years (32–43)	38 years (33–43)
Age category		
18–29 years	52 (13%)	47 (13%)
30–44 years	260 (65%)	253 (67%)
45+ years	76 (20%)	87 (22%)
Non–South African nationality	75 (19%)	66 (18%)
Unemployed	95 (24%)	64 (17%)
On FDC	356 (89%)	331 (88%)
Time on ART, median (IQR)	1.9 years (1.6–2.4)	1.9 years (1.6–2.3)
Hypertensive	21 (5%)	27 (7%)
Baseline CD4, median (IQR)	472 cells/mm^3^ (342–665)	527 cells/mm^3^ (377–690)
CD4 category		
<350 cells/mm^3^	108 (27%)	80 (21%)
350–499 cells/mm^3^	101 (25%)	92 (25%)
≥500 cells/mm^3^	188 (47%)	204 (54%)

Data are *n* (percent) unless otherwise indicated.

ART, antiretroviral therapy; FDC, fixed-dose combination; IQR, interquartile range.

### Follow-up and outcomes

**Fig 3:**
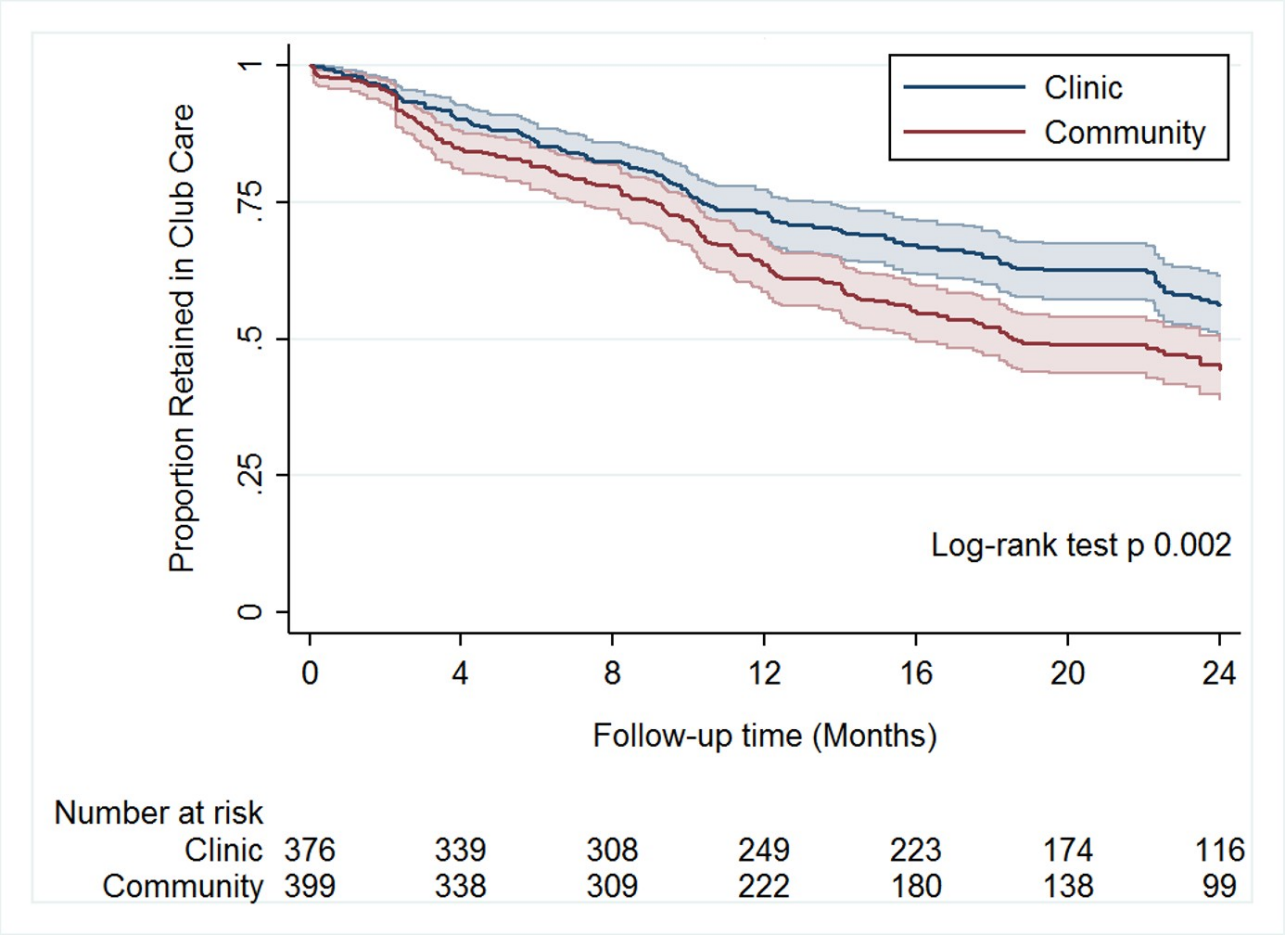
Kaplan–Meier curves of loss to club-based care by treatment arm (*n =* 775). Cumulative proportion retained in club-based care, by study arm. The shading around each plot represents the 95% CI.

Over 24 months of follow-up (until 31 May 2017), 5,878 person-months were accumulated by those assigned to community-based clubs, and 6,323 by those assigned to clinic-based clubs. A total of 367 (47%, 95% CI 44%–51%) participants experienced the primary outcome of loss from club-based care. The intraclass correlation coefficient with respect to clustering of outcomes by study arm was 0.04 (95% CI 0.00–0.75). Among community-based club participants, the cumulative proportion lost from club-based care by 2 years was 52% (95% CI 47%–57%), compared to 43% (95% CI 38%–48%, *p =* 0.002) among clinic-based club participants (see Fig 3), a difference of 9% (95% CI 2%–16%, *p =* 0.012). In the primary analysis, after adjusting for sex, age, nationality, time on ART, employment status, and baseline CD4 count, the risk of loss to club-based care was 38% higher among participants assigned to community-based clubs than among those assigned to clinic-based clubs (adjusted hazard ratio [HR] 1.38, 95% CI 1.02–1.87, *p =* 0.032). In a secondary analysis, a univariate Cox proportional hazards model, this estimate remained similar (HR 1.39, 95% CI 1.02–1.89, *p =* 0.035). In a post hoc analysis, after adjusting for sex, age, nationality, time on ART, employment status, and baseline CD4 count, the adjusted risk difference in the cumulative proportion lost from club-based care (community-based club arm minus clinic-based club arm) was 9% (95% CI 0%–19%, *p =* 0.077).

Among those who were lost from club-based care (*n =* 367), the most common reason was missing a club visit and the associated ART pickup entirely (*n =* 198, 54%, 95% CI 49%–59%) (see Table 3). This proportion was higher, but not significantly so, among those lost from community-based club care (58%, 95% CI 51%–65%) as compared with those lost from clinic-based club care (49%, 95% CI 41%–57%, *p =* 0.086). Documented viral rebound was higher, again not significantly so, among participants assigned to clinic-based clubs (21%, 95% CI 13%–27%) than among those assigned to community-based clubs (13%, 95% CI 8%–18%, *p =* 0.051). Voluntary return to clinic-based standard care was uncommon (7% overall, 95% CI 4%–10%), and was similar between treatment arms (*p =* 0.429). No participants in either treatment arm died during club-based care.

**Table 3:** Reasons for loss from club-based care by treatment arm (*n =* 367).

Reason for loss from club-based care	Community-based clubs (*n =* 207)			Clinic-based clubs (*n =* 160)			*p-*Value
	*n*	Percent	95% CI	*n*	Percent	95% CI	
Missed club visit and ART pickup	120	58	51–65	78	49	41–56	0.079
Viral rebound	27	13	8–18	33	21	14–27	0.051
Pregnancy	16	8	4–11	20	13	7–18	0.128
Other club rule violation	20	10	5–15	16	10	6–14	0.914
Voluntary return to standard of care	16	8	4–11	9	6	2–9	0.429
Developed excluding comorbidity^1^	6	3	1–5	4	3	0.8–5	0.816
Regimen change	2	1	0.3–3	0	0	—	0.212
Death (all cause)	0	0	—	0	—	—	—

^1^Details on excluding comorbidities—community-based arm: uncontrolled glucose (*n =* 3), renal impairment (*n =* 2), mental health diagnosis (*n =* 1); clinic-based arm: tuberculosis (*n =* 2), renal impairment (*n =* 1), uncontrolled glucose (*n =* 1).

ART, antiretroviral therapy.

Among the 367 participants lost to club-based care, 266 (72%, 95% CI 68%–77%) reengaged in clinic-based care within 90 days of their club-based care discontinuation date. This proportion was similar by treatment arm (72% among community-based participants [95% CI 66%–79%] and 73% among clinic-based participants [95% CI 66%–79%], *p =* 0.993).

**Fig 4:**
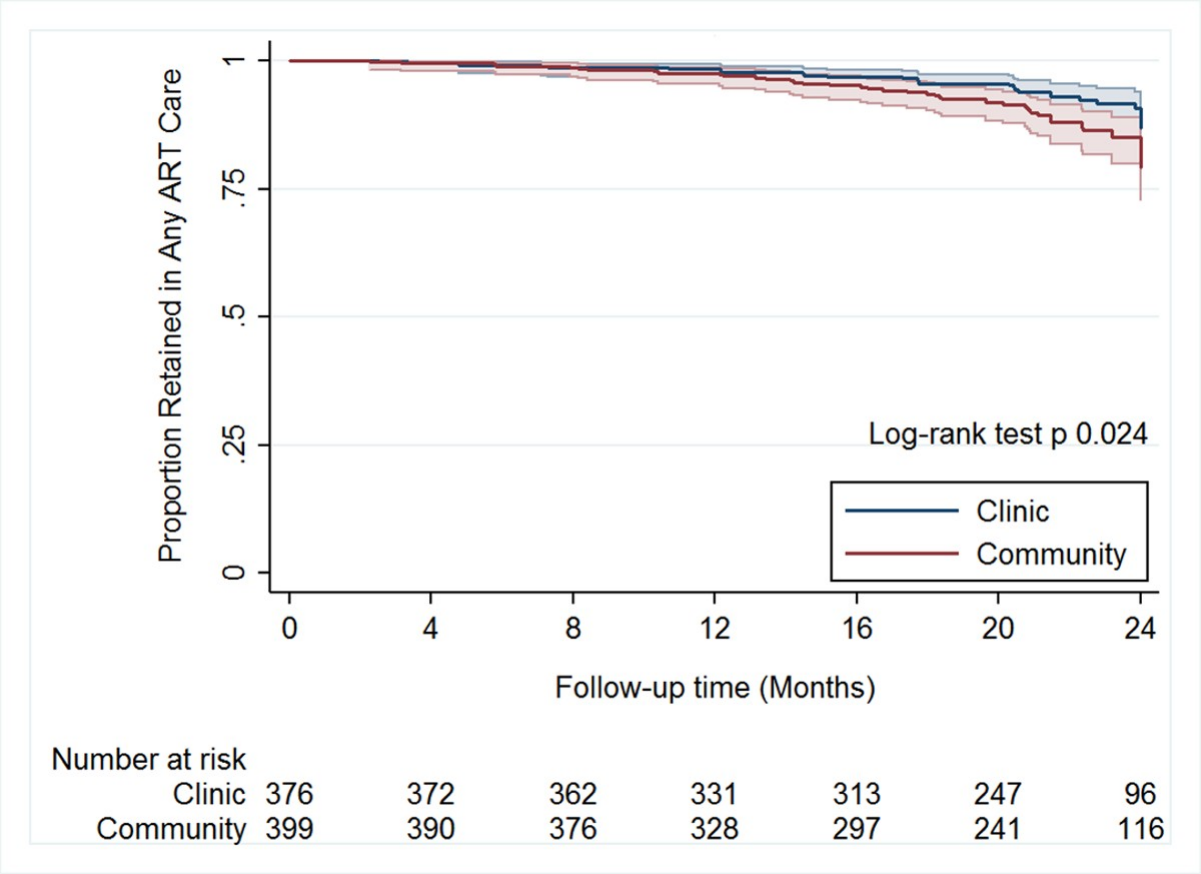
Kaplan–Meier curves of loss to any ART care (club-based or clinic-based standard care) by treatment arm (*n =* 775). Cumulative proportion retained in any ART care, by study arm. The shading around each plot represents the 95% CI. ART, antiretroviral therapy.

We also examined a secondary outcome of loss from any ART care (either club-based care or clinic-based standard care following discontinuation from club-based care). Overall, 77 (10%, 95% CI 8%–12%) participants were lost from ART care during follow-up. Among community-based club participants, the proportion lost from any ART care was 12% (95% CI 9%–16%), compared to 7% (95% CI 5%–10%, *p =* 0.024) among clinic-based club participants (see Fig 4), corresponding to a difference of 5% (95% CI 1%–9%, *p =* 0.018). In a univariate Cox proportional hazards model, the risk of loss to any ART care was non-significantly increased among participants assigned to community-based clubs as compared with those assigned to clinic-based clubs (HR 1.69, 95% CI 0.98–2.91, *p =* 0.057). In a sensitivity analysis, after adjusting for baseline differences in sex, age, nationality, time on ART, employment status, and baseline CD4 count, this estimate was similar (adjusted HR 1.65, 95% CI 0.96–2.83, *p =* 0.068).

## Discussion

Findings from this pragmatic randomized controlled trial demonstrate that loss from an adherence club intervention for stable patients on ART in South Africa was high overall, with only 53% of all participants virally suppressed and retained in club care at 24 months following the first club visit. Importantly, loss from club-based care was significantly higher among those in community-based clubs (52%) compared to those in clubs based within the clinic (43%). Although the majority (72%) of adherence club participants who were referred back to clinic-based standard care reengaged in care within 90 days, the disparity between participants in community- versus clinic-based clubs persisted when considering the outcome of 24-month loss from any kind of ART care. Such poor adherence club retention, where nearly half of those receiving the intervention were referred back into routine clinic-based care, cannot be considered a success, particularly given that decongesting busy clinics and streamlining patient care are the primary goals of adherence clubs.

These findings are in contrast to those from 2 published studies examining the effectiveness of adherence clubs from Cape Town, South Africa, in which retention in ART care for adherence club participants was excellent (94% at 12 months [8] and 97% at 18 months [9]) and represented an improvement over receiving ART according to the routine standard of care. Crucially, neither study from Cape Town reported on the outcome of retention within club-based care, choosing instead to focus on retention in any ART care (inclusive of both club-based care and routine clinic-based standard care). Retention in club-based care is an important outcome to consider, as the identification and recruitment of patients for participation in adherence clubs and the logistical considerations of organizing clubs and hosting club visits requires significant clinic resources, including clinician and pharmacist time, which are not efficiently utilized if nearly half of patients return to routine clinic-based care within 2 years of starting the intervention. Study design could also explain contrasting findings between this study and those previously published from Cape Town. This study randomized participants, stratified by their area of residence, to community- or clinic-based clubs. The Cape Town studies, by contrast, were both observational cohort studies and thus subject to selection bias, where those participants who are recruited and choose the intervention may differ in important ways with respect to factors related to retention in care from those who are not recruited. This is underscored in the methods of Luque-Fernandez et al.: “Only some stable patients were offered participation [in adherence clubs], based on the clinician’s enthusiasm for the model” [9]. Randomized trials of health services interventions, such as adherence clubs, can provide a high-quality evidence base for understanding their effectiveness and utility, and are especially important as differentiated care approaches are developed and implemented for different populations of people living with HIV. We look forward to forthcoming findings from a cluster randomized trial of the South African National Adherence Guidelines for Chronic Diseases, including ART adherence clubs, which will examine 14-month viral suppression and retention in care compared to the routine standard of care [11].

The most common reason in this study for loss from both community and clinic club-based care was missing a club visit and its associated medication pickup, while voluntary withdrawal and viral rebound were relatively uncommon in both arms. Data presented here do not illuminate why patients missed club visits and did not pick up their medication; however, we can hypothesize that it may be a complicated mixture of convenience (e.g., needing to see clinicians outside of club-based care), scheduling logistics (e.g., rigidity of club schedule), and concerns around stigma or confidentiality. For some participants in this study, the perceived cost of receiving ART care through adherence clubs may outweigh the perceived value, resulting in disengagement from care [12]. For example, a patient whose work schedule is not regular (e.g., who works casually in “piece jobs”) may experience a conflict in having work available on the day of a scheduled adherence club. The patient may weigh the value (i.e., attending the club and continuing to receive the benefit and convenience of club-based care) with the cost (i.e., loss of a day’s worth of wages), and decide that, although more time-consuming, receiving clinic-based standard care is ultimately more flexible, given the unpredictability of work opportunities. Typically, within the routine clinic-based standard of care, there is little downside to skipping a clinic appointment (if such appointment systems even exist), and many experienced ART patients may maintain a stockpile of medication to bridge short-term gaps [13]. Other models of differentiated care such as ART delivered in vending machines or at fast-track pharmacy queues may offer less stringent rules and thus more flexibility for some patients. We are in the process of analyzing the data of a mixed-methods evaluation of the acceptability of clinic- and community-based adherence clubs, which will be published separately; however, a preliminary analysis found a preference for clinic-based clubs owing to reasons of stigma and access to additional health services [14]. We anticipate these results may inform improvements on these care models.

This study has several limitations. Follow-up was limited to 24 months as a proxy for what is intended to be a lifelong intervention, and thus estimates of loss from club-based care may underestimate that which occurs over a longer span of time. Those dropping out of care at the study clinic may have sought care at other clinics despite not being transferred out, and thus we may have overestimated loss from any ART care. Treatment assignment was unblinded for practical reasons, but could have led to differential loss from club-based care between treatment arms. Aside from death, we did not collect specific data on other potential adverse events (e.g., social harms as a result of the intervention), although data on comorbidities were routinely collected. Finally, this study was conducted at a single clinic in a high-burden, middle-income setting in urban South Africa, and thus its findings may not be generalizable to settings where a lower HIV burden, fewer resources, or more rural geography may introduce additional implementation challenges that may not make the intervention feasible in such settings.

In summary, in the present study, our findings demonstrate that losses from clinic-based adherence clubs were lower compared to those from community-based adherence clubs. We urge caution in assuming that the effectiveness of clinic-based interventions will carry over to the community setting, without a better understanding of patient-level factors associated with loss from care. Examining the outcome of loss from club-based care rather than just loss from ART care is essential to understanding the value of these and future interventions designed to provide differentiated care to people living with HIV. Differentiated care models for ART will undoubtedly evolve going forward, and it will be necessary to understand differences in patient preferences and perspectives in order to maximize beneficial outcomes for all patients. As ART availability increases globally, it will be important to efficiently deliver reliable and effective methods of treatment in order to meet ambitious global targets of viral suppression in 90% of those initiated on ART [15].

## Supporting information

S1 Data Data files sufficient to replicate analyses. (CSV)

S1 Text Study protocol. (PDF)

S2 Text CONSORT checklist. (DOC)

## Abbreviations

ART: antiretroviral therapy
HR: hazard ratio
TB: tuberculosis
WHO: World Health Organization
