# Supplementary material for: The impact of community- versus clinic-based adherence clubs on loss from care and viral suppression for antiretroviral therapy patients: Findings from a pragmatic randomized controlled trial in South Africa
Source: PLoS Med. 2019 May 21;16(5):e1002808. doi: 10.1371/journal.pmed.1002808 (PMC6528966; doi:10.1371/journal.pmed.1002808)
Supplement: S1 Text — (PDF) [file pmed.1002808.s002.pdf]

Community Versus Clinic-based ART Adherence Clubs to Enhance Patient Long-term Retention-in-Care

Executive Summary

Adherence clubs are groups of 25-30 patients stable on antiretroviral treatment (ART) who meet once every two months with club facilitators (counselors, and nurses on dates of blood draws) for a one hour session during which counseling is provided and ART medication distributed. Clinic-based adherence clubs implemented in Khayelitsha, South Africa have demonstrated that patients participating in clubs have reduced loss to follow up and lower risk of viral load rebound compared to stable patients receiving routine clinic-based care. Key questions remain as to whether the effectiveness of adherence clubs differs when based at the clinic compared to those based in the community. We propose an unblinded randomized controlled trial of community-based versus clinic-based adherence clubs for patients who are stable on ART. The overall goal of this study is to evaluate whether, compared to clinic-based adherence clubs, community-based clubs result in improved patient outcomes and greater retention in care without compromising overall quality of patient medical care.

Participating Agencies and Investigators

Witkoppen Health and Welfare Center, Johannesburg, South Africa

Valerie Keyser, MBChB – Principal Investigator
Nora West, MPH – Research Programs Manager

University of North Carolina at Chapel Hill, Chapel Hill, United States

Annelies Van Rie, MD, PhD, Associate Professor, Department of Epidemiology
Colleen Hanrahan, PhD, Postdoctoral Researcher, Department of Epidemiology
Sheree Schwartz, PhD, Postdoctoral researcher, Department of Epidemiology

Funding Source

USAID Innovations Grant AID-674-A-12-00033

Version 1.1 17 July 2014

Abbreviations

ANC	Antenatal care
ART	Antiretroviral therapy
FGD	Focus Group Discussion
GCP	Good clinical practices
LTFU	Loss to follow-up
PHCN	Primary health care nurse
PI	Principal investigator
SOC	Standard of care
VL	Viral load
WHWC	Witkoppen Health and Welfare Centre

1. Introduction

South Africa has the largest antiretroviral therapy (ART) programme in the world. Since the roll-out of the ART programme in 2004, AIDS related deaths have continued to decline in South Africa and prevalence has stabilized. At the end of 2010 it was estimated that 1.4 million people were receiving ART in South Africa, which represented an estimated coverage of 55% of persons eligible for treatment [1]. Since that time South African treatment guidelines have continued to expand to include individuals with higher CD4 counts. In the era of treatment as prevention, efforts will continue to expand access and uptake of treatment, however successful approaches will need to minimize costs and maximize retention in care.

Task-shifting is one mechanism through which to expand coverage and maintain more patients in care. Two randomized controlled studies from South Africa have demonstrated that nurse managed and/or initiated ART care is not inferior in terms of patient outcomes compared to care provided by medical doctors [2, 3]. Nurse-managed care makes ART available to more people as it expands the number of health care workers available to initiate ART. Additional task-shifting measures to decentralize care to the community may be able to further reduce the queues in clinics and the burden on providers.

Successful ART programmes require high coverage of patient initiation on ART, but also high rates of treatment success (i.e. virological suppression) and long-term retention in care. A systematic review of retention outcomes in sub-Saharan Africa estimated that 75% of patients initiated onto ARVs between 2000-2007 were retained in care at 12 months, decreasing to 62% by 24 months [4]. While a follow-up review from 2007-2009 demonstrated some improvement as 77% remained in care at 24 months and 70% at 36 months [5], these figures highlight that additional measures are needed to maximize long-term retention on ART.

The adherence club model task-shifts HIV care and makes services more patient-friendly. This model was first tested in the Cape Town township of Khayelitsha by Médicins Sans Frontières. The Khayelitsha model offered patients who were virologically suppressed and stable on their ARV treatment participation in adherence clubs. Clubs were organized as groups of 25-30 stable HIV-infected patients who met once every two months with club facilitators (counselors, and nurses on dates of blood draws) for a one hour session during which counseling and ART was distributed. The investigators reported a 57% reduction in loss to follow-up (LTFU) and a 67% decrease in viral load rebound in adherence club participants compared to patient receiving standard of care [6].

Several key questions remain. First, as this study was observational, it is unclear whether there were differences between those who chose to join clubs and those who did not. Second, it remains unknown if the effectiveness depends on whether the club is based at the clinic or in the community. Community-based adherence clubs may be even more patient friendly as they require less transport by the patient and do not require scarce clinic space. However stigma and fear of seeking care close to one's home may be barriers to community-based clubs.

2. Overall Goal and Objectives

The overall goal of this study is to evaluate whether community-based versus clinic-based adherence clubs are a patient friendly intervention which result in improved patient outcomes and greater retention in care without compromising overall quality of ART care.

2.1. Primary objective

Assess the effectiveness of community-based versus clinic-based adherence clubs to retain patients who are stable on ART in care and to maintain HIV viral suppression over a two year period.

Hypothesis: A higher proportion of patients in community-based clubs will have improved retention in care and persistent HIV viral suppression relative to patients in clinic-based adherence clubs.

2.2. Secondary objectives

Perform comparisons between community-based and clinic-based adherence clubs of

- The proportion of patients who choose to stop adherence club participation and return to standard of care clinic based services
- The proportion of patients with contra-indications for continuation of adherence club participation
- The proportion of patients identified with new or previously undiagnosed medical conditions at their annual (clinic-based) doctor visit
- The uptake of routine clinic services including family planning, cervical cancer screening and ANC care
- The acceptability of adherence clubs
- Twenty-four month (2-year) all-cause mortality

3. Methods

3.1. Study Setting

Witkoppen Health and Welfare Centre (WHWC) is a high-volume primary health care clinic in northern Johannesburg, and primarily serves Diepsloot, an area of formal and informal settlements. WHWC sees on average 8,500 patients a month, of whom about 40% are HIV-infected. The clinic has currently started clinic-based adherence clubs on a pilot basis, and has established four clubs of approximately 30 patients each who meet every other month at the clinic for a one-hour adherence club visit. These pilot clubs are not part of this proposed study, however operational lessons learned were used to develop the proposed study club structure and data collection tools.

3.2. Club locations

Clinic-based clubs will be located onsite at WHWC. Community-based clubs will be located in 6-8 sites throughout the local communities of Kya Sands, Diepsloot, Msawawa, and Cosmo City. The facilities where community-based clubs will meet vary and include churches, non-governmental organizations, community based organizations and local government facilities. Locations are specifically chosen to ensure sufficient geographic coverage of the WHWC catchment area. Additionally, facilities have been intentionally selected to minimize HIV-related stigma as facilities are multi-purpose and used by various groups at different times throughout the week for meetings which may be community-related and health or non-health related. Locations were selected in consultation with the community service providers and the WHWC community advisory forum.

3.3. Study Design Overview

This study is an unblinded randomized controlled trial of community-based versus clinic-based adherence clubs in WHWC patients who are stable on ART defined as in section 3.3.1. Patients will be screened for adherence club eligibility, those eligible will be informed about the study, and those consented will be randomized to a clinic-based or community-based adherence club. Individuals will be grouped into clubs by their area of residence to ensure that those randomized to community-based clubs will require minimal travel to the club venue. Clubs will meet every two months to provide adherence counseling and distribute ART. Patients will be followed for outcomes for twenty-four months following their first club visit. Data will be collected through review of club registers, electronic record review, clinic file review and an annual acceptability questionnaire.

3.3. Study Population

The study population will be drawn from individuals receiving routine ART care at WHWC, and residing in the areas of Diepsloot, Cosmo City, Kya Sands and Msawawa.

Each club will have a minimum of 25 participants, and will be capped at 30 participants. A minimum of 600 participants will be enrolled into the study (25-30 participants in each of 24 clubs) and a maximum of 800 (the maximum may be greater than 720 as additional participants may be admitted from waiting lists if clubs fall below 20).

3.4. Inclusion/Exclusion Criteria

3.4.1. Inclusion Criteria

- Age ≥18 years
- No change in ART regimen in the past year, OR a regimen change ≥ 6 months prior to eligibility screening in the following circumstances: when (a) the patient in question has been changed from Zidovudine to Tenofovir, and/or Nevirapine to Efavirenz, and/or Stavudine to Zidovudine or Tenofovir, and (b) their most recent creatinine level was normal
- Virally suppressed for a least 12 months. Patients will be considered eligible if their two most recent (pre-baseline and baseline) viral load results were <400 copies/ml, and the baseline viral load is <200 copies/ml.

3.4.2. Exclusion Criteria

- On D4T containing regimen (in order ensure optimal management of side effects)
- Currently pregnant, or intending to become pregnant within 6 months at the formation of the club
- Current comorbidity or chronic illness (diabetes, epilepsy, active TB, cancer, mental illness, or other disease which requires routine and frequent clinical management).
- Hypertension which requires treatment with on more than one antihypertensive drug

3.5. Recruitment and enrolment

Recruitment for adherence clubs will be conducted on a monthly rolling basis, with the goal of launching at least one pair (one clinic-based and one community-based) of clubs per month. For each month of recruitment, a geographic target area of residence will be pre-determined. Patients attending WHWC who are currently on ART will be pre-screened by clinicians during their routine medical visits at the recruitment visit (Visit 0, Table 1). Clinicians will follow the Medical Screening Form (See Appendix 1) in order to determine eligibility. Patients who are eligible will be approached for enrolment and will have a baseline blood draw to confirm VL suppression if interested. Consenting patients will be enrolled and if they live in the geographic target area for that month, will be randomized to a clinic-based or community-based club. Participants who consent to join adherence clubs but reside outside of the geographic target area for the month will either be randomized to a club for an upcoming month in which that area is targeted, or will be entered into a database and contacted at the start of the month that club recruitment for their area of residence is planned. Patients on the waiting list longer than 1 month will have a baseline VL repeated prior to their first adherence club visit.

Figure 1: Recruitment and enrolment flow at Visit 0

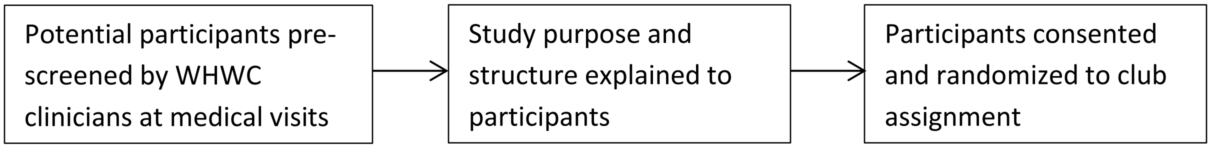

Clubs will have a minimum of 25 participants, and will be capped at 30 participants. Eligible patients who are recruited after a monthly club has been filled will be assigned to the next available club if the geographic area is suitable, or entered into a database and contacted to return for study enrolment when a suitable club becomes available. Participants on the waiting list may also be slotted into on-going clubs which have dropped below 20 participants, provided the geographic area of the club is appropriate. Results of baseline bloods collected at enrolment will be reviewed by the Retention in Care Coordinator. Participants with (1) a baseline VL >400 copies/ml OR (2) a baseline blood of 50-400 copies/ml AND a previous VL also in this range will be contacted, excluded from further study participation and reassigned to receive standard of care at the clinic. Retention, clinical and laboratory results will be collected for patients who successfully completed pre-screening, consented to participate, and were enrolled into the study prior to determination of club ineligibility per their baseline viral load.

A minimum of 600 participants will be enrolled into the study (25-30 participants in each of 24 clubs) and a maximum of 800 (the maximum may be greater than 720 as additional participants may be admitted from waiting lists if clubs fall below 25).

3.6. Study procedures

3.6.1. Recruitment visit

The recruitment visit (Visit 0, Table 1) is conducted at a patient's routine medical visit to WHWC. Patients who are identified by clinicians as eligible for an adherence club will be referred to the Retention in Care Coordinator who will explain the concept of adherence clubs, and if the patient is interested, will consent the patient to join the study. The coordinator will thoroughly explain the randomised club assignment prior to consent. Following consent, the patient will be referred to a study assistant for random club assignment and a baseline blood draw.

3.6.2. Randomisation

Eligible participants residing in a target geographic area will be randomly assigned to either a clinic-based or community-based adherence club. After the eligible patient has consented to study participation, a study research assistant (separate from the Retention in Care Coordinator) will perform the randomised assignment of club location. Participants will choose an opaque envelope containing a randomly allocated club assignment. Participants cannot switch club assignment after randomization.

3.6.3. Club visits

The first club visit (Visit 1, Table 1) for all participants will be conducted at the clinic. All club participants will meet at the clinic at the appointed day and time. All subsequent club visits for community-based clubs will be conducted at the allocated community site or at the clinic for clinic-based clubs.

Each club visit will be attended by at least one club counselor, who will run the club. Participant attendance will be recorded. Each patient will be verbally screened for current TB symptoms (current cough of any length, current fever, unintentional weight loss and/or night sweats). Patients experiencing any TB symptom will be referred to the clinic to give a sputum specimen. Patients will be weighed, and then will meet briefly to discuss an adherence or ART-related topic, led by the club counselor. Each patient will receive their two month pre-packed supply of medication. Each adherence club visit should last approximately one hour. After year 1, the cycle of club visits repeats, starting with Visit 1.

The counselor will measure the blood pressure of hypertensive patients at the start of each club visit, and record them in the register. Any abnormal reading (Systolic>140 or Diastolic>100 mm) will be repeated at the end of the club visit. Any participant with 2 abnormal readings at the same club visit will be referred for clinician evaluation at WHWC.
Participants can have a “buddy” pick up their medication at adherence club visits. The buddy is pre-selected by the participant and documented upon enrolment. The counselor in charge of the club will verify the buddy’s identification, and have the buddy sign for receipt of the participant’s medication. The participant may not send a buddy for two consecutive club visits.

Table 1: Years 1 and 2 adherence club visits and activities

YEAR 1								
**Visit**	**0**	**1**	**2**	**3**	**4**	**5**	**6**	**7**
**Month**	**-1**	**0**	**2**	**4**	**6**	**8**	**10**	**12**
**Type of visit**	Recruitment & Screening	Club visit *AT CLINIC*	Club visit	Club visit	Club visit	Club visit	Club visit	Medical visit *AT CLINIC*
**Activities**	Consent and Baseline blood draw	Routine	Blood draw for VL 50-400 copies;	Routine	Rescripting	Routine	Blood draw for all	Medical review and rescripting Acceptability questionnaire

YEAR 2						
**Visit**	**8**	**9**	**10**	**11**	**12**	**13**
**Month**	**14**	**16**	**18**	**20**	**22**	**24**
**Type of visit**	Club visit	Club visit	Club visit	Club visit	Club visit	Medical visit *AT CLINIC*
**Activities**	Blood draw for VL 50-400 copies;	Routine	Rescripting	Routine	Blood draw for all	Medical review and rescripting Acceptability Questionnaire

3.6.4. Blood draws and re-scripting of medication

We will collect 1mL of blood at the enrolment visit in order to check the HIV viral load.
Blood draws will be conducted at clinic and community-based club visits as indicated in Table 1 by a PHCN. The blood draw at Visit 2 is restricted to only patients who had a VL of 50-400 copies/ml on their baseline blood draw. The blood draw at Visits 6 and 22 is for all patients, in preparation for their annual medical review at Visits 7 (month 12) and 13 (month 24). At each blood draw, 1 mL of blood will be taken in order to check the HIV viral load.
Rescripting of ART will occur twice annually at club Visits 4,7,10 and 13. At visits 4 and 10, this will be done at the clinic or community-based club visit by the PHCN. At Visits 7 and 13, the annual medical review, this will be done by the clinic-based WHWC clinician.

3.6.5. Acceptability questionnaire

A brief (5-10 minute) questionnaire covering participant acceptability of adherence clubs will be conducted annually at club Visits 7 and 13 (see Table 1 and Appendix 2). Questionnaires will be

administered at the end of the club visit, and will be conducted individually, using a team of 4-5 interviewers in order to expedite the process.

3.6.6. Criteria for discontinuation of adherence club participation

Participants can be returned to clinic-based standard-of-care (SOC) at any point for the following reasons:

- **Voluntary return, i.e.** participants who decide they prefer SOC over adherence clubs
- **Two consecutive "buddy" pickups in a row**.
- **Two consecutive late pickups or three late pick-ups in 12 months:** If a participant cannot pick up their medication at a club visit and does not send a buddy, they can pick it up from the Retention in Care Coordinator at WHWC within 5 days of the club visit.
- **Missing a medication pickup**, defined as a patient who fails to present for the adherence club, does not send a buddy, and does not collect his/her drugs at WHWC within 5 days of scheduled club visit.
- **Pregnancy:** Participants reporting pregnancy will be referred to the clinic for consultation and confirmation. Similar to weight loss above, a clinician will determine whether the patient must be returned to SOC. Patients may return to clubs one year postpartum.
- **TB diagnosis:** If a TB diagnosis is made, this participant will be returned to SOC. Participants may return to clubs upon successful completion of TB treatment.
- **Hypertensives:** Participants who are referred back to the clinic with abnormal blood pressure will be assessed by a clinician, and the clinician will determine whether the participant should be returned to SOC. All participants requiring treatment with more than one anti-hypertensive will be returned to SOC.
- **Identification of excluding comorbid or chronic condition** at the annual medical visit or at any other incidental clinic visit during the year.
- **Viral rebound or persistent viral "blips"** defined as one VL >400 OR a VL of <200 followed by a next VL>50 copies/ml.
- **ART regimen change** for any reason (side effects, abnormal safety bloods or viral rebound). Patients who have a regimen change due to side effects or abnormal safety bloods will receive SOC but may return to adherence clubs after one year of viral suppression on the new regimen.

The Retention in Care Coordinator will be responsible for managing the transition of patients who are no longer eligible for adherence clubs into SOC by booking clinic appointments and liaising with the WHWC clinician on the participant's clinical history. Patients who do not present for SOC will be traced using the routine WHWC defaulter tracing program.

3.6.7. Patient follow-up and data collection

The total follow-up time for all participants will be twenty-four months following the first club visit. Data on participants will be collected from several sources:

- **Club register** (Appendix 3)
  Club registers will be maintained by the counselor leading each club and will include a roster of attendees, weight, TB symptom screening results, "buddy" pickup, late or missed pickup.
- **Annual acceptability questionnaire** (Appendix 2)
  At Visits 7 and 13 (Table 1), study staff will administer a short questionnaire covering patient acceptability of the clinic and community-based adherence clubs.
- **Electronic data collection** (Appendix 4)

Patient laboratory data (VL, CD4 count and safety bloods including creatinine clearance, GFR, AST/ALT and full blood count) will be collected from Therapy Edge or TrakCare, two electronic laboratory databases used by WHWC.

- **Paper file review** (Appendix 4)
  Participant clinic files will be reviewed in order to abstract baseline and updated demographic data and clinical data including co-morbidity history, pregnancy and visits to the clinic during study follow-up.
- **In-depth interviews (IDIs)** (Appendix 5)
  After first year of study completion study investigators and the Retention in Care Coordinator will facilitate audio-recorded IDIs to explore strengths and limitations of the community versus club based models.

3.6.8. In-depth interviews

After the first year of study participation, one-on-one in-depth interviews (IDIs) will be held with 24 participants. Twelve IDIs will be held with members of on-site clubs and 12 with members of community-based clubs. Each session will last around 60 minutes and will be comprised of individuals who are enrolled in the study. Participants will be purposefully selected based on their type of club, gender and their attendance records in order to ensure diversity of the participants. Participation is voluntary and will require a separate consent process; there will be no penalty or loss in standing to participants who cannot or choose not to participate in the IDIs. Timing of the interviews will occur after the first year in order to better understand from the club attendees what the challenges are of attending club visits at the clinic versus in the community, and to better understand the participants' perspectives on which model of care if preferable and why. Patient names will not be used in the interviews in order to protect participant confidentiality. IDIs will take place separately from club visits at WHWC and transport costs will be reimbursed.

3.6.9. Roles and responsibilities

Table 2: Roles and responsibilities of study and clinical staff

Role	Responsibility
Retention in Care Coordinator	Coordinate patient recruitment, enrolment and participant consent, lead clubs, supervise club counselors, follow up on individuals symptomatic for TB, review baseline and follow-up blood results for eligibility, assist in annual acceptability questionnaire administration, coordinate return to routine clinic-based care, coordinate late pickups and monitor continued participant eligibility, facilitate transition to SOC
Study PI	Supervise on all aspects of study, consult on challenging eligibility screens, consult on baseline and follow-up blood draw review
WHWC clinicians	Screen patients for club eligibility, conduct annual medical visit, rescript ART
Research data assistants	Perform informed consent and randomised allocation of club assignment, compile monthly statistics from club registers, perform file reviews for clinical follow-up, assist in annual acceptability questionnaire administration
Club counselor	Lead community and clinic-based clubs
Club PHCN	Perform necessary blood draws and rescripting ART

3.7. Sample size

The primary outcome of this study is the combined 24-month outcome of viral rebound or LTFU, as this represents the two main goals of the intervention: retention in care and maintenance of viral suppression through ART adherence. Viral rebound is defined as having a VL >400 copies/ml, while retention in care is defined being actively retained in adherence club-based care. Our primary hypothesis is that participants in community-based clubs will experience less viral rebound and LTFU relative to patients in clinic-based adherence clubs. Table 3 presents a range of proportions of the combined outcome for each arm of the study, and the resulting *total* sample size required to detect this difference between arms, assuming 90% power, a two-sided α of 0.05 and equal sample size among each arm. Assuming that we recruit two clubs of at least 25 members per month, after one year of enrolment we can achieve a total sample size of at least 600 individuals (300 in each arm). At this sample size, we will be able to detect an absolute improvement in the outcome of at least 10% in the community-based clubs compared to clinic-based clubs.

Table 3: Total sample size reflecting 90% power to detect a decrease in the proportion of community-based adherence club participants experiencing viral rebound or LTFU compared to those in clinic-based adherence clubs

		LTFU/VL Rebound Community-based clubs			
		**5%**	**10%**	**15%**	**20%**
**LTFU/ VL Rebound Clinic-based clubs**	**10%**	1242	-	-	-
	**15%**	**414**	1914	-	-
	**20%**	**228**	**572**	2504	-
	**25%**	**150**	**292**	708	3008

1.1. Data Management

1.1.1. Data collection

Data from club visits will be collected on a standardised club register (see Appendix 3). Likewise, the annual acceptability questionnaire will be conducted using a standardized structured questionnaire (see Appendix 2). Participant clinic file reviews will be conducted annually using a standardised abstraction form (see Appendix 4). Each form will be entered into an electronic Access database using a unique participant identifier, the WHWC file number in order to link records from individuals. A linkage file between participant identifiers and the study number will be kept separately from all other data. Electronic data abstraction from Therapy Edge and TrakCare will be performed annually. In-depth interviews will be conducted after the first year of the study (see Appendix 5).

1.1.2. Data confidentiality

All study staff will be trained in good clinical practice, which emphasizes the principles of strict data confidentiality. Each enrolled participant will be referenced using their unique WHWC file number. File numbers and names will be used on the club registers as the study is part of the clinic's service delivery and are necessary to ensure that notes, scripts and other data is kept up-to-date in the patients' files, and to prevent errors in distributing medication. All materials containing patient identifiers will be kept in a locked cabinet within a locked room when not in active use, and only study staff will have access to these materials. Data, excluding names, will be captured in an electronic password protected Access database to which only authorised study staff has access, and will be stored on a secure server. Any data transfer between WHWC and UNC will be done using the secure file server, Sonus, which is a

password protected, encrypted file transfer system. Data transfers will never include any participant identifiers. Once the database is closed for analysis, WHWC file numbers will be permanently deleted from the database, and participants will only be assigned a unique study ID.

1.2. Data Analysis

Standardized descriptive statistics will be used to characterise the two study arms. We will explore the relationship between the exposure of interest, community-based versus clinic-based adherence club assignment on the primary outcome of viral rebound or LTFU using multivariate Cox Proportional Hazards models, adjusting for age, sex, time on ART, nationality and other potentially confounding factors. We will graphically explore this relationship using Kaplan Meier survival curves. We will utilize multivariate Poisson regression in order to examine the relationship between club assignment and the outcome of number of clinic visits outside of the adherence club schedule. We will use multivariate logistic regression in order to examine the relationship between club assignment and other individual participant characteristics with the outcome of uptake of other routine wellness services for HIV infected patients on ART. The acceptability questionnaire will be translated into a numeric acceptability index, and we will examine the relationship between club assignment and other individual participant characteristics with this index using multivariate linear regression.

1.3. Dissemination and publication of results

Results of the study will be disseminated to stakeholders including but not limited to WHWC, University of the Witwatersrand, USAID, the Gauteng provincial Department of Health, Johannesburg Region A Department of Health, and the National Department of Health. Results may also be presented at relevant national and international scientific meetings.

2. Ethical Considerations

2.1. Informed consent and enrolment

Patients at WHWC who are currently on ART will be screened by clinicians for adherence club eligibility (see Appendix 1). Eligible patients will be referred to the GCP trained Retention in Care Coordinator who will who will explain the concept of adherence clubs, and if the patient is interested, will consent the patient to join the study. Informed consent will be conducted in private in the patient's choice of English, Zulu or Sesotho. After the patient and Retention in Care Coordinator have thoroughly explained the purpose of the study, all study procedures including the randomization of club assignment and discussed any questions or concerns, the patient will choose to provide written consent or to decline participation in the study (see Appendix 6). Participants who are unable to read will have the consent form read aloud to them and can then sign with a thumbprint or mark to indicate their consent. For these participants, a witness will be present for the consent process. A copy of the consent form will be offered to the study participant to keep. A separate consent process for participation in in-depth interviews will be performed as described above.

After the participant has given informed consent, they will be referred to a study research assistant who will perform the randomisation of club assignment.

2.2. Risks and benefits of participation

2.2.1. Benefits

Participants of this study will directly benefit by having the opportunity to participate in adherence clubs. Clubs will not be offered to patients outside of study participation. The clubs benefit participants by providing a streamlined process for ART medication pickup that lasts approximately one hour. Current clinic wait times for ART pickup can be as long as 4-6 hours. Additionally, for participants who receive assignment to a community-based club, the club will be

located within their community of residence, which may be more convenient than travelling to WHWC for medication pickup.

2.2.2. Risks

Patients who participate in this study will be eligible to receive all necessary medical care at WHWC regardless of the type of club assignment they receive. Participants who are assigned to community-based clubs could be less likely to seek necessary medical care outside of club visits compared to those attending clinic-based clubs simply because the participant is not already at the clinic, where choosing to see a clinician if needed is more convenient. Participants in both study arms will be encouraged to attend WHWC for any medical needs outside of ART medication, including routine clinic services. Blood results will still be collected per the frequency of national guidelines and TB and weight loss screening will occur to ensure timely diagnosis of new illnesses or complications.

This study involves collection of routine clinical information. Disclosure of this information could be potentially damaging to the study participants. These risks will be minimised by ensuring that all study team members are trained in Good Clinical Practices and re-emphasising the principles surrounding confidentiality on a regular basis in order to ensure that clinical information is handled with respect and in line with the highest research ethics standards. Additionally, rigorous measures will be taken in order to ensure confidentiality of participant data (see 1.1.2 Data confidentiality). By signing the consent form, all participants pledge to keep the identity of other participants and all information shared at clubs in strict confidence. Preselected buddy's who pick up participant medication will be required to sign for receipt of the medication as well as pledge to keep the identity of participants and details of the clubs confidential. All community-based sites will sign confidentiality agreements regarding patients who attend clubs at their facilities. Furthermore community sites have been identified with the local community and have been selected because of their diversity of services such that visiting the site is not an indication to the community of HIV status.

2.2.3. Compensation

Participants in the main study will not be compensated for participation. Participants completing the in-depth interviews will be compensated R50 for transportation to and from WHWC.

3. Study Timeline

Activity	Q4 2013	Q1/2 2014	Q3/4 2014	Q1/2 2015	Q3/4 2015	Q1/2 2016	Q3/4 2016	Q1/2 2016
Ethics approval	■							
Staff training	■							
Participant enrolment		■	■					
Participant follow up		■	■	■	■	■	■	
In-depth interviews				■				
Data management			■	■	■	■	■	■
Data analysis						■	■	■

Results dissemination								

4. References

1. World Health Organization. Global HIV/AIDS response: epidemic update and health sector progress towards universal access: progress report 2011.
2. Sanne I, Orrell C, Fox MP, Conradie F, Ive P, Zeinecker J*, et al.* Nurse versus doctor management of HIV-infected patients receiving antiretroviral therapy (CIPRA-SA): a randomised non-inferiority trial. *Lancet* 2010,**376**:33-40.
3. Fairall L, Bachmann MO, Lombard C, Timmerman V, Uebel K, Zwarenstein M*, et al.* Task shifting of antiretroviral treatment from doctors to primary-care nurses in South Africa (STRETCH): a pragmatic, parallel, cluster-randomised trial. *Lancet* 2012,**380**:889-898.
4. Rosen S, Fox MP, Gill CJ. Patient retention in antiretroviral therapy programs in sub-Saharan Africa: a systematic review. *PLoS Med* 2007,**4**:e298.
5. Fox MP, Rosen S. Patient retention in antiretroviral therapy programs up to three years on treatment in sub-Saharan Africa, 2007-2009: systematic review. *Trop Med Int Health* 2010,**15 Suppl 1**:1-15.
6. Luque-Fernandez MA, Van Cutsem G, Goemaere E, Hilderbrand K, Schomaker M, Mantangana N*, et al.* Effectiveness of patient adherence groups as a model of care for stable patients on antiretroviral therapy in Khayelitsha, Cape Town, South Africa. *PLoS One* 2013,**8**:e56088.

APPENDIX 1: Medical Screening Form

WHWC Adherence Clubs – Medical Screening Form

**TE Sticker**	**Screening visit 1**<br>___ ___/___ ___/___ ___<br>d d m m y y		**Bloods Review**<br>___ ___/___ ___/___ ___<br>d d m m y y	
**Virally suppressed for last 12 months**<br>*Two most recent viral load results <400 copies/ml, with no more than one result >200 copies/ml*<br>☐LDL ☐50-400	☐Yes	**CONTINUE SCREENING**	**Virally suppressed?**	
	☐No	STOP **NOT ELIGIBLE**	☐LDL<br>☐50-400	☐ >400<br>☐50-400
**Female AND Currently pregnant**	☐No	**CONTINUE SCREENING**	CONTINUE	STOP
	☐Yes	STOP **NOT ELIGIBLE**	**Safety bloods OK?**	
**Female AND Intend on getting pregnant in next 6 months**	☐No	**CONTINUE SCREENING**	☐Yes	☐No
	☐Yes	STOP **NOT ELIGIBLE**	**ENROL**	STOP
**On current regimen for ≥ 1 year or ≥ 6 months when changed from AZT->TFV; NVP->EFV; D4T->TFV or AZT**	☐Yes	**CONTINUE SCREENING**		
	☐No	STOP **NOT ELIGIBLE**		
**On D4T**	☐No	**CONTINUE SCREENING**		
	☐Yes	STOP **NOT ELIGIBLE**		
**Currently experiencing side effects**	☐No	**CONTINUE SCREENING**		
	☐Yes	STOP **NOT ELIGIBLE**		
**Current TB Suspect or on TB Treatment?**	☐No	**CONTINUE SCREENING**		
	☐Yes	STOP **NOT ELIGIBLE**		
**Unstable hypertensive AND/OR on more than 1 anti-hypertension treatment?** *To be eligible, most recent BP must be less than 140/90.*	☐No – not HYT	**CONTINUE SCREENING**		
	☐No – stable on 1 trtmnt	**CONTINUE SCREENING**		
	☐Yes	STOP **NOT ELIGIBLE**		
**Any excluding co-morbidities?**<br>DIABETES, HEART DISEASE, CANCER, EPILEPSY, HEPATITIS, CHRONIC MENTAL ILLNESS, ASTHMA, OTHER CHRONIC ILLNESS	☐No	**CONTINUE SCREENING**		
	☐Yes	STOP **NOT ELIGIBLE**		
**Currently wants to attend clinic visits with positive child**	☐No	**CONTINUE SCREENING**		
	☐Yes	STOP **NOT ELIGIBLE**		
**Any other medical reason to exclude?**<br>**Specify:**________________________________<br>____________________________________	☐No	**ELIGIBLE**		
	☐Yes	STOP **NOT ELIGIBLE**		
**Patient refused/uninterested due to:**	**ELIGIBLE?**			

☐ Work conflict<br>☐ Prefers attending clinic at WHWC<br>☐ Is currently fast-tracked at WHWC<br>☐ Other: ________________________________	• 1 mo+5 repeats for ARVs and HYT medication (if applicable)<br>• Send to Linda for enrolment<br>• Send for VL and CD4 count	

APPENDIX 2: Acceptability Questionnaire

Participant ID: ____ ____ - ____ ____ ____ ____

Date: ____/_____/__________
dd mm yyyy

Adherence Club Questionnaire

We would like to ask you some questions about your experience participating in adherence clubs. All the information will be kept confidential and will not be shared with anyone else besides the research study staff. Please feel free to be honest and open.

What your feelings on the following things about adherence clubs:

a. Location of club	Like	Neither like nor dislike	Dislike
b. Length of club visits	Too short	Just right	Too long
c. Time of day the club is held	Too early	Time is ok	Too late
d. Size of club	Too small	Size is ok	Too big
e. Quality of counseling / support received during club visits	Good quality	Quality is fair	Poor quality

Do you have any of the following worries about participating in adherence clubs?

	Yes	No	If yes, describe
a. Concern over transportation to the location of the club			
b. Concern over level of individual attention received during club visits			
c. Concern about not seeing a doctor or nurse more frequently			
d. Concern over confidentiality during club visits			
e. Concern over confidentiality because of club location			
f. Other concerns experienced (specify)			

Overall, how do you feel about participating in adherence clubs?

- ☐ Like
- ☐ Neither like nor dislike
- ☐ Dislike

Any additional feedback about your experiences in the clubs:

____

____

____

APPENDIX 3: Club Register

APPENDIX 4: File review fields

Information to be collected through file review (electronic review on Therapy Edge or paper-based clinic file)

**Demographics**
• Age<br>• Sex (M/F)
**Clinical history**
• ART initiation date<br>• Current ART regimen<br>• Date started current ART regimen<br>• CD4 nadir & CD4 nadir date<br>• Last CD4 & date of last CD4<br>• Last two VL prior to study screening & dates<br>• History of TB<br>• Weight at enrolment
**Health during study**
• CD4 counts & dates throughout study<br>• VL & dates throughout study<br>• Blood pressure at enrollment & follow-up medical visit(s)<br>• Co-morbid conditions throughout study<br>• GFR & creatinine at enrolment & follow-up<br>• Family planning at enrolment & follow-up medical visit(s)<br>• Incident pregnancy<br>• Change in HAART regimen (rx & date)
**Clinic visits**
• Number & type of non-club visits to WHWC during FU<br>• Description of any event, illness, diagnosis or condition that returned participant to regular care at clinic

APPENDIX 5: In-depth Interview Guide

WITKOPPEN HEALTH & WELFARE CENTRE

ADHERENCE CLUB IN-DEPTH INTERVIEW GUIDE TO BE ADMINISTERED AT END OF YR1

Welcome and thank you for coming today. You have been asked to participate in this interview because we want to learn more about the experiences you have all had participating in adherence clubs. Our goal is to understand what has worked well for you and what could be improved.

Please indicate by circling whether participants below to: clinic-based club or community-based club

1. **Tell us about your experience belonging to an adherence club.**

   Probes:

   - *What did you like about participating in the clubs?*
   - *What didn't you like about participating in the clubs?*
   - *Have there been any good things that have happened to you – or someone else you know who is in an adherence club run by Witkoppen Health and Welfare Centre– because of participation in the adherence clubs?*
   - *Have there been any bad things or events that have happened to you --or someone else participating in the Witkoppen adherence clubs – because of participation in the adherence clubs?*

2. **If a friend was interested in participating in the adherence clubs and asked you for advice, what would you tell them based on your experience?**

3. Where do you think that the adherence clubs should be located – at the clinic or in the community? Why?

Probes:

- *What are the advantages to having the adherence clubs run on-site at Witkoppen Clinic?*
- *What are the disadvantages to having the adherence clubs run on-site at Witkoppen Clinic?*
- *What are the advantages to having the adherence clubs run in the community?*
- *What are the disadvantages to having the adherence clubs run in the community?*

4. How do you think that your experience participating in an adherence club would have been different if your club had been based in the [clinic/community] instead of in the [community/clinic]?

Probes:

- *Overall do you think that it would have been better, worse or about the same?*
- *Do you think that you would be more likely to skip club visits if they are held at the clinic or in the community?*

5. **Would you continue participating in your club if it were moved to the [clinic/community]? Why or why not?**

6. **Would you continue participating in your club if it were run by the Department of Health instead of Witkoppen, but the services and care stayed the same? Why or why not?**

7. **Did you like where you club was located in the [community/clinic]?**

   Probes:

   - *What made the location of your site good?*
   - *What made the location of your site bad or problematic?*
   - *What problems did you or your adherence club face that were related to the location of where your clubs were held?*
   - *Did others in the community know why you were going to the site? Did they know you were part of an HIV adherence club?*
   - *How do you feel about community members or people you know seeing you go the location where the adherence club was held?*
   - *Where would you suggest that the clubs be held within the community?*
